# Physical Therapy in Wound Healing, Edema, and Urinary Incontinence

**DOI:** 10.1155/2014/825826

**Published:** 2014-05-11

**Authors:** Jakub Taradaj, Tomasz Urbanek, Luther C. Kloth, Marco Romanelli

**Affiliations:** ^1^Department of Physiotherapy Basics, Academy School of Physical Education in Katowice, Mikolowska 72 Street, 40-065 Katowice, Poland; ^2^Department of General and Vascular Surgery, Medical University of Silesia in Katowice, Ziolowa 45 Street, 40-635 Katowice, Poland; ^3^Department of Physical Therapy, Marquette University, Schroeder Complex 346, Milwaukee, WI 53201-1881, USA; ^4^Department of Dermatology, University of Pisa, Via Roma 67, 56126 Pisa, Italy

The development of civilization leads to many chronic diseases. Of the health problems specific to frail both young and older people, unhealed chronic wounds (venous and pressure ulcers and diabetic foot), cancer-related lymphedema, and urinary incontinence are the major health disorders, and the establishment and spread of effective treatment methods for the following health problems are a pressing issue. The described disorders are a common and costly problem in nursing home settings, with the prevalence of estimates varying widely from 17 to even 53%.

Care and management can have significant economic consequences. Staff time for ongoing assessment, documentation, and dressing changes and expensive pharmaceuticals drain the available resources. Well-documented, promising, and inexpensive methods for physical therapy are necessary.

This special issue includes eight interesting papers. It has to be mentioned that this issue contains, among others, the following main topics: new promising methods in wound healing, prevalence, diagnostics, surgery, and physical therapy of urinary incontinence, electromyography and biofeedback in rehabilitation of pelvic floor muscles, and kinesiology taping in lymphedema.

Each manuscript submitted to the issue underwent during the course of the peer-review process by three independent researchers. The peer-review process was single blinded; that is, the reviewers knew who the authors of the manuscript are, but the authors did not have access to the information of who the peer reviewers are.

We believe that published articles will be interesting for readers.

*Jakub Taradaj* *Tomasz Urbanek* *Luther C. Kloth* *Marco Romanelli*

